# Investigating the Association Between Semaglutide Use and Mood Disorders in Polyendocrine Metabolic Ovarian Syndrome: A Retrospective Cohort Study​

**DOI:** 10.7759/cureus.114569

**Published:** 2026-08-15

**Authors:** Freida P Raj, Harry Boo, Ashley Kenney, Eduardo Espiridion

**Affiliations:** 1 Obstetrics and Gynecology, Drexel College of Medicine, Philadelphia, USA; 2 Psychiatry, Drexel University College of Medicine, Reading, USA; 3 Psychiatry, Texas Christian University, Fort Worth, USA; 4 Psychiatry, Reading Hospital Tower Health, West Reading, USA

**Keywords:** generalized anxiety disorder (gad), glp-1 agonist, insomia, major depressive disorder (mdd), pcos and metabolic syndrome, polycystic ovary syndrome (pcos), polyendocrine metabolic ovarian syndrome, suicidal ideations

## Abstract

Objective: This retrospective cohort study aims to evaluate whether semaglutide use in patients with polyendocrine metabolic ovarian syndrome (PMOS) is associated with an increased risk of mood disorders compared to patients treated with metformin by utilizing a large, propensity-matched dataset from TriNetX.

Methodology: The TriNetX platform was used to conduct a retrospective cohort study with International Classification of Diseases, 10th Revision (ICD-10) codes to generate inclusion and exclusion criteria. After propensity matching, each cohort consisted of 20,535 patients, for a total sample size of 41,070. Cohort 1 (patients with PMOS and on metformin) and Cohort 2 (patients with PMOS and on semaglutide) were evaluated for outcomes of new-onset major depressive disorder, generalized anxiety disorder, insomnia, and suicidal ideation that emerged after the start of the medication of interest.

Results: There was no statistically significant difference in generalized anxiety disorder and suicidal ideation associated with semaglutide use as compared to metformin in women with PMOS. However, there was a minor statistically significant difference in the rates of insomnia and major depressive disorder.

Conclusions: These results suggest that psychiatric considerations alone should not preclude the use of semaglutide in PMOS management. Ongoing mental health screening and follow-up remain essential for all patients with PMOS, regardless of treatment choice.

## Introduction

Polyendocrine metabolic ovarian syndrome (PMOS), formerly known as polycystic ovary syndrome (PCOS), is an endocrine disorder associated with hyperandrogenism, metabolic disorder, and ovarian dysfunction [1]. While treating PMOS is often aimed at reducing these clinical symptoms, individuals with PMOS also face a higher risk of mood disorders. One recent meta-analysis found that patients with PMOS are three times as likely to have depressive symptoms compared with individuals of the same age group [2]. Moreover, the prevalence of depression in patients with PMOS ranges between 16% and 55%, compared to just 2.6% to 5.9% in the general population. Patients with PMOS are also at higher risk of sleep disturbances. A meta-analysis of nine studies involving more than 1,000 participants found that PMOS was positively associated with sleep disturbances and was associated with lower sleep quality scores across multiple validated assessment tools [3].

Currently, the treatment aims of PMOS are targeted toward managing and preventing potential metabolic dysregulation and the symptoms of hyperandrogenism and ovarian dysfunction, such as acne, irregular menstruation, and hirsutism. The American Society for Reproductive Medicine recommends that women with PMOS be regularly screened for cardiovascular disease, type 2 diabetes, and obstructive sleep apnea [4]. More specifically, the American College of Obstetrics and Gynecology recommends screening for type 2 diabetes mellitus using a glucose challenge rather than a hemoglobin A1C measurement in the general population [5]. Individuals with PMOS should also be screened for anxiety and depression with validated tools, alongside disordered eating and psychosexual dysfunction as deemed appropriate [4].

Combined oral contraceptives are a first-line treatment for menstrual irregularity and hyperandrogenism by suppressing luteinizing hormone secretion and ovarian androgen secretion [5]. However, lifestyle modifications and the use of antidiabetic drugs have shown efficacy not only in the management of metabolic syndrome, but also in improving ovarian function. Moreover, metformin has been shown to increase ovulation in women with PMOS with odds ratios of 3.88 compared with placebo, according to one meta-analysis [6]. More recently, there has been an emergence and popularization of glucagon-like peptide-1 (GLP-1) receptor agonist medications for the treatment of type 2 diabetes and obesity. This class of medications works to delay gastric emptying, suppress appetite, and increase the feeling of satiety - all of which lead to reductions in body mass index (BMI) and greater glycemic control [7]. For use in PMOS, one meta-analysis found that women with both PMOS and obesity who were treated with GLP-1 agonists had reductions in BMI, waist circumference, and testosterone levels - this indicates that GLP-1 medications are a viable treatment option for managing PMOS in women with comorbid obesity [8]. However, these medications come with a risk of adverse gastrointestinal events, such as nausea, vomiting, and, more rarely, pancreatitis. Moreover, GLP-1 receptor agonists have also been linked to potential mental health effects. One recent study looked at the perception of semaglutide users through social media posts and found that individuals reported weight loss led to either a significant improvement or deterioration in mood [9]. Furthermore, users frequently mentioned side effects such as anxiety and insomnia.

The current literature establishes that PMOS is associated with an increased risk of mood disorders and metabolic dysfunction. Semaglutide and other GLP-1 receptor agonists have demonstrated efficacy in addressing metabolic and weight-related outcomes among patients with PMOS. However, the relationship between semaglutide use and psychiatric outcomes in this population remains poorly characterized, particularly in comparison with established treatments such as metformin. Although previous studies have examined the metabolic benefits of GLP-1 receptor agonists in PMOS, limited research has evaluated whether semaglutide use is associated with differential risks of depression, anxiety, insomnia, and suicidal ideation. This gap is clinically relevant given the high prevalence of psychiatric comorbidities among individuals with PMOS and the increasing use of GLP-1 receptor agonists for metabolic management. Therefore, this retrospective cohort study evaluates whether semaglutide use among patients with PMOS is associated with differences in psychiatric outcomes compared with metformin using a large, propensity-matched dataset from TriNetX [10].

## Materials and methods

The TriNetX platform was used to conduct a retrospective cohort study [10]. The inclusion and exclusion criteria were outlined using ICD-10 codes [10]. Since the ICD-10 code for PMOS does not yet exist given the recent name change, the PCOS ICD-10 code was used for the data analysis. Cohort 1 consisted of patients with PCOS/PMOS (E28.2) who were on metformin; patients who took both metformin and semaglutide were excluded. Cohort 2 consisted of patients with PCOS/PMOS (E28.2) who were on semaglutide; patients who took both semaglutide and metformin were excluded. Medication exposure was defined based on documented medication records within TriNetX. Patients were included if the assigned treatment (metformin or semaglutide) was documented continuously for the full one-year study period. Patients with documented switching to the alternative treatment or without continuous documentation of the assigned treatment for the full year were excluded.

The outcomes evaluated included major depressive disorder (MDD) (F33), generalized anxiety disorder (GAD (F41.1), insomnia (F51), and suicidal ideation (R45.851). The mood outcomes were evaluated after the start of the medication of interest, either metformin or semaglutide. All patients with a history of MDD, GAD, insomnia, and suicidal ideation were excluded. The study period for this dataset was December 2025.

Data source

TriNetX is a global health research platform that aggregates de-identified electronic medical records from a global collaborative network [10]. This study analyzed patients from 166 healthcare organizations. TriNetX allows researchers access to comprehensive clinical data, including demographics, diagnoses, medications, procedures, and laboratory values [10].

Participants

The participants in this study included de-identified data from 166 healthcare organizations. The two cohorts created for this study were propensity-matched based on age, race, ethnicity, and BMI. Age, race, and ethnicity were included to account for demographic differences that may influence treatment selection, healthcare utilization, and the prevalence or recognition of mental health conditions. Baseline BMI was included as a matching variable because it is closely linked to PMOS severity, healthcare utilization, and the underlying risk of depression, anxiety, and sleep disturbances. Additionally, higher BMI strongly influences treatment selection, as patients with greater adiposity are more likely to be prescribed semaglutide. Matching on BMI therefore reduced treatment-selection bias, minimized confounding related to baseline obesity, and improved comparability between groups when evaluating mood and insomnia outcomes. After propensity matching, each cohort consisted of 20,535 patients, with a total sample size of 41,070. The control cohort consisted of PMOS patients on metformin, and the comparative cohort consisted of PMOS patients on semaglutide.

Statistical methods

The TriNetX “Compare Cohorts” analysis function was used to evaluate the association and survival between the two cohorts [10]. For each outcome, absolute risk, risk difference (RD), risk ratio (RR), odds ratio (OR), 95% confidence intervals (CIs), and corresponding p-values were calculated. Z-tests were used to evaluate risk difference and risk ratio estimates, whereas chi-square statistics were reported for odds ratio analyses. Time-to-event outcomes were analyzed using Kaplan-Meier survival analysis, with differences between survival curves assessed using the log-rank test. Hazard ratios (HRs) and 95% CIs were estimated using Cox proportional hazards regression. Statistical significance was defined as a two-sided *P*-value < 0.05.

## Results

Demographics

Patients from each cohort were propensity-matched based on age, race, and ethnicity, ensuring compatibility between the two cohorts. Each cohort included 20,535 patients aged 18 to 65 years (Table 1).

**Table 1:** Characteristics of patients with PMOS who were on either metformin or semaglutide after matching cohorts on age, race, ethnicity, and BMI (n = 20,535 per cohort). The table describes the demographic characteristics of patients in the analysis. 1 = Cohort with PMOS who were prescribed metformin (*n* = 20,535). 2 = Cohort with PMOS who were prescribed semaglutide (*n* = 20,535). Age is presented as mean ± standard deviation (SD), while categorical variables are shown as percentages. Std. Diff. refers to standardized difference. TriNetX matches patients using a propensity score function by assigning each patient a propensity score for each given covariate, then pairing patients for each covariate with their nearest propensity score neighbor. Standardized difference is the quantifiable difference in the means of a covariate between two groups, divided by the pooled standard deviation. The propensity score function sets a default caliper of 0.1, such that the standardized difference for each covariate must be below the threshold of 0.1 to be considered adequately matched. PMOS, polyendocrine metabolic ovarian syndrome

Cohort	Demographics	Mean ± SD	Patients (*n*)	% of cohort	Std. diff.
1	Current age	38.2 ± 10.5	20,535	100%	0.025
2		38.5 ± 10.4	20,535	100%	
1	Age at index	36.3 ± 10.3	20,535	100%	0.022
2		36.5 ± 10.2	20,535	100%	
1	White		14,833	72.2%	0.006
2			14,779	72.0%	
1	American Indian or Alaska Native		107	0.5%	0.014
2			128	0.6%	
1	Unknown Race		1,382	6.7%	<0.001
2			1,380	6.7%	
1	Native Hawaiian or Other Pacific Islander		74	0.4%	0.031
2			117	0.6%	
1	Unknown Ethnicity		3,723	18.1%	0.005
2			3,760	18.3%	
1	Not Hispanic or Latino		14,587	71.0%	0.006
2			14,646	71.3%	
1	Hispanic or Latino		2,225	10.8%	0.015
2			2,129	10.4%	
1	Black or African American		2,661	13.0%	0.016
2			2,774	13.5%	
1	Other race		874	4.3%	0.013
2			819	4.0%	
1	Asian		604	2.9%	0.020
2			538	2.6%	

Baseline BMI distributions across different BMI ranges were also matched between cohorts (Table 2).

**Table 2:** Baseline BMI characteristics after propensity score matching. This table summarizes the distribution of body mass index (BMI, kg/m²) in the propensity score-matched cohorts. Cohort 1 consists of patients with PMOS prescribed metformin (*n* = 20,535), and Cohort 2 consists of patients with PMOS prescribed semaglutide (*n* = 20,535). BMI categories are presented as the number and percentage of patients in each cohort to demonstrate the balance achieved after propensity score matching. “Std. Diff.” refers to standardized difference. TriNetX matches patients using a propensity score function by assigning each patient a propensity score for each given covariate, then pairing patients for each covariate with their nearest propensity score neighbor. Standardized difference is the quantifiable difference in the means of a covariate between two groups, divided by the pooled standard deviation. The propensity score function sets a default caliper of 0.1, such that the standardized difference for each covariate must be below the threshold of 0.1 to be considered appropriately matched. PMOS, polyendocrine metabolic ovarian syndrome

Cohort	BMI	Patients	% of Cohort	Std. Diff.
1	<20	106	0.5%	0.046
2		186	0.9%	
1	20.0-20.9	30	0.10%	0.012
2		21	0.10%	
1	21.0-21.9	39	0.20%	0.005
2		35	0.20%	
1	22.0-22.9	62	0.30%	0.013
2		48	0.20%	
1	23.0-23.9	91	0.40%	0.018
2		68	0.30%	
1	24.0-24.9	115	0.60%	0.005
2		108	0.50%	
1	25.0-25.9	188	0.90%	0.001
2		189	0.90%	
1	26.0-26.9	187	0.90%	0.025
2		238	1.20%	
1	27.0-27.9	261	1.30%	0.028
2		329	1.60%	
1	28.0-28.9	293	1.40%	0.044
2		411	2.00%	
1	29.0-29.9	347	1.70%	0.061
2		527	2.60%	
1	30.0-30.9	552	2.70%	0.097
2		923	4.50%	
1	31.0-31.9	571	2.80%	0.11
2		1,006	4.90%	
1	32.0-32.9	661	3.20%	0.092
2		1,037	5.00%	
1	33.0-33.9	695	3.40%	0.109
2		1,160	5.60%	
1	34.0-34.9	738	3.60%	0.107
2		1,202	5.90%	
1	35.0-35.9	854	4.20%	0.129
2		1,462	7.10%	
1	36.0-36.9	820	4.00%	0.118
2		1,362	6.60%	
1	37.0-37.9	809	3.90%	0.139
2		1,461	7.10%	
1	38.0-38.9	801	3.90%	0.126
2		1,382	6.70%	
1	39.0-39.9	783	3.80%	0.125
2		1,350	6.60%	
1	40.0-44.9	2,539	12.40%	0.224
2		4,232	20.60%	
1	45.0-49.9	1,631	7.90%	0.194
2		2,867	14.00%	
1	50.0-59.9	1,129	5.50%	0.181
2		2,129	10.50%	
1	60.0-69.9	323	1.60%	0.084
2		576	2.80%	
1	≥70	90	0.40%	0.057
2		186	0.90%	

MDD

Within the first year of being given their first prescription, MDD occurred in 345 of 18,160 (absolute risk of 0.019) patients in the metformin group and 385 of 17,406 (absolute risk of 0.022) patients in the semaglutide group, amounting to a risk difference of -0.003 (95% CI: -0.006 to -0.000; *z* = -2.075; *P* = 0.038) and a risk ratio of 0.859 (95% CI: 0.744-0.992) (Table 3).​​​​​

**Table 3:** Measures of absolute MDD outcomes for patients with PMOS within one year of being prescribed either metformin or semaglutide. This table shows the absolute number of patients with PMOS who had a first diagnosis of MDD within one year of being prescribed either metformin or semaglutide. Survival probabilities were estimated using the Kaplan-Meier method as implemented in the TriNetX platform. Hazard ratios were calculated using Cox proportional hazards regression. A total of 2,375 patients in Cohort 1 and 3,129 patients in Cohort 2 were excluded from the analysis because they had been diagnosed with MDD before their prescription. MDD, major depressive disorder; PMOS, polycystic metabolic ovarian syndrome

Cohort	Patients in cohort	Patients with outcome	Risk	Survival probability
Metformin	18,160	345	0.019	97.47%
Semaglutide	17,406	385	0.022	97.28%

The Kaplan-Meier survival curve analysis showed that patients in the metformin group had a 97.47% survival probability with respect to developing MDD, compared to 97.28% for the semaglutide group. Accordingly, the hazard ratio for MDD comparing metformin users to semaglutide users was 0.925 (95% CI: 0.800, 1.070). The log-rank test was (χ² = 1.104, df = 1, *P* = 0.293) (Table 4).

**Table 4:** Measures of comparison in MDD outcomes for patients with PMOS within one year of being prescribed either metformin or semaglutide. This table presents risk measures for major depressive disorder (MDD) within one year, comparing patients with PMOS prescribed metformin versus semaglutide. Risk difference (RD) was calculated as the absolute difference in cumulative incidence between cohorts, risk ratio (RR) as the ratio of cumulative incidence, and odds ratio (OR) from the corresponding 2 × 2 contingency table. Hazard ratios (HRs) were estimated using a Cox proportional hazards regression model, while Kaplan-Meier survival curves were compared using the log-rank test. Corresponding *z*-statistics, chi-square statistics, *P*-values, and 95% confidence intervals (CIs) are reported for the appropriate analyses to indicate the statistical significance and precision of each estimate. Collectively, these measures quantify differences in the one-year risk of MDD between the metformin and semaglutide cohorts. PMOS, polycystic metabolic ovarian syndrome

Metric	Value	95% CI	z	*P*-value	Chi-squared	df
Risk difference	-0.003	-0.006 to -0.000	-2.075	0.038	-	-
Risk ratio	0.859	0.744-0.992	-	-	-	-
Odds ratio	0.856	0.739-0.992	-	-	-	-
Hazard ratio	0.925	0.800-1.070	0.069	0.793	0.069	1
Log-rank rest	-	-	-	0.293	1.104	1

GAD

Within the first year of being given their first prescription, GAD occurred in 472 of 17,497 (absolute risk of 0.027) patients in the metformin group and 456 of 16,479 (absolute risk of 0.028) patients in the semaglutide group, amounting to a risk difference of -0.001 (95% CI: -0.004 to 0.003; *z* = -0.393; *P* = 0.694) and a risk ratio of 0.975 (95% CI: 0.859-1.107) (Table 5).

**Table 5:** Measures of absolute GAD outcomes for patients with PMOS within one year of being prescribed either metformin or semaglutide. This table shows the absolute number of patients with PMOS who had a first diagnosis of GAD within one year of being prescribed either metformin or semaglutide. Survival probabilities were estimated using the Kaplan-Meier method as implemented in the TriNetX Analytics platform. Hazard ratios were calculated using Cox proportional hazards regression. A total of 3,038 patients in Cohort 1 and 4,056 patients in Cohort 2 were excluded from the analysis because they had been diagnosed with GAD before their prescription. GAD, generalized anxiety disorder; PMOS, polycystic metabolic ovarian syndrome

Cohort	Patients in cohort	Patients with outcome	Risk	Survival probability
Metformin	17,497	472	0.027	96.41%
Semaglutide	16,479	456	0.028	96.57%

The Kaplan-Meier survival curve analysis showed that patients in the metformin group had a 96.41% survival probability with respect to developing GAD, compared to 96.57% for the semaglutide group. Accordingly, the hazard ratio for GAD comparing metformin users to semaglutide users was 1.049 (95% CI: 0.923-1.194). The log-rank test was (χ² = 0.003, df = 1, *P* = 0.462) (Table 6).​​​​​​​

**Table 6:** Measures of comparison in GAD outcomes for patients with PMOS within one year of being prescribed either metformin or semaglutide. This table presents risk measures for generalized anxiety disorder (GAD) within one year, comparing patients with PMOS prescribed metformin versus semaglutide. Risk difference (RD) was calculated as the absolute difference in cumulative incidence between cohorts, risk ratio (RR) as the ratio of cumulative incidence, and odds ratio (OR) from the corresponding 2 × 2 contingency table. Hazard ratios (HRs) were estimated using a Cox proportional hazards regression model, while Kaplan-Meier survival curves were compared using the log-rank test. Corresponding *z*-statistics, chi-square statistics, *P*-values, and 95% confidence intervals (CIs) are reported for the appropriate analyses to indicate the statistical significance and precision of each estimate. Collectively, these measures quantify differences in the one-year risk of GAD between the metformin and semaglutide cohorts. PMOS, polycystic metabolic ovarian syndrome

Metric	Value	95% CI	z	*P*-value	Chi-squared	df
Risk difference	-0.001	-0.004 to 0.003	-0.393	0.694	-	-
Risk ratio	0.975	0.859-1.107	-	-	-	-
Odds ratio	0.974	0.855-1.110	-	-	-	-
Hazard ratio	1.049	0.923-1.194	-	0.955	0.003	1
Log-rank test	-	-	-	0.462	0.540	1

Sleep disorder/insomnia

Within the first year of being given their first prescription, sleep disorders/insomnia occurred in 208 of 19,676 (absolute risk of 0.011) patients in the metformin group and 249 of 19,165 (absolute risk of 0.013) patients in the semaglutide group, amounting to a risk difference of -0.002 (95% CI: -0.005 to -0.000; *z* = -2.121; *P* = 0.027) and a risk ratio of 0.814 (95% CI: 0.678-0.977) (Table 7).​​​​​​​

**Table 7:** Measures of absolute sleep disorder/insomnia outcomes for patients with PMOS within one year of being prescribed either metformin or semaglutide. This table shows the absolute number of patients with PMOS who had a first diagnosis of sleep disorder/insomnia within one year of being prescribed either metformin or semaglutide. Survival probabilities were estimated using the Kaplan-Meier method as implemented in the TriNetX Analytics platform. Hazard ratios were calculated using Cox proportional hazards regression. A total of 859 patients in Cohort 1 and 1,370 patients in Cohort 2 were excluded from the analysis because they had been diagnosed with sleep disorders/insomnia before their prescription. PMOS, polycystic metabolic ovarian syndrome

Cohort	Patients in cohort	Patients with outcome	Risk	Survival probability
Metformin	19,676	208	0.011	98.61%
Semaglutide	19,165	249	0.013	98.39%

The Kaplan-Meier survival curve analysis showed that patients in the metformin group had a 98.61% survival probability with respect to developing sleep disorders/insomnia, compared to 98.39% for the semaglutide group. Accordingly, the hazard ratio for sleep disorders/insomnia comparing metformin users to semaglutide users was 0.872 (95% CI: 0.726-1.049). The log-rank test was (χ² = 2.112, df = 1, *P* = 0.146) (Table 8)​​​​​​​.

**Table 8:** Measures of comparison in sleep disorder/insomnia outcomes for patients with PMOS within one year of being prescribed either metformin or semaglutide. This table presents risk measures for sleep disorder/insomnia within one year, comparing patients with PMOS prescribed metformin versus semaglutide. Risk difference (RD) was calculated as the absolute difference in cumulative incidence between cohorts, risk ratio (RR) as the ratio of cumulative incidence, and odds ratio (OR) from the corresponding 2 × 2 contingency table. Hazard ratios (HRs) were estimated using a Cox proportional hazards regression model, while Kaplan-Meier survival curves were compared using the log-rank test. Corresponding *z*-statistics, chi-square statistics, *P*-values, and 95% confidence intervals (CIs) are reported for the appropriate analyses to indicate the statistical significance and precision of each estimate. Collectively, these measures quantify differences in the one-year risk of sleep disorder/insomnia between the metformin and semaglutide cohorts. PMOS, polycystic metabolic ovarian syndrome

Metric	Value	95% CI	z	*P*-value	Chi-squared	df
Risk difference	-0.002	-0.005 to -0.000	-2.212	0.027	-	-
Risk ratio	0.814	0.678-0.977	-	-	-	-
Odds ratio	0.812	0.674-0.977	-	-	-	-
Hazard ratio	0.872	0.726-1.049	-	0.415	0.663	1
Log-rank test	-	-	-	0.146	2.112	1

Suicidal ideation

Within the first year of being given their first prescription, suicidal ideation occurred in 73 of 19,887 (absolute risk of 0.004) patients in the metformin group and 56 of 20,030 (absolute risk of 0.003) patients in the semaglutide group, amounting to a risk difference of 0.001 (95% CI: -0.000 to 0.002; *z* = -1.540; *P* = 0.124) and a risk ratio of 1.313 (95% CI: 0.927-1.859) (Table 9).

**Table 9:** Measures of absolute suicidal ideation outcomes for patients with PMOS within one year of being prescribed either metformin or semaglutide. This table shows the absolute number of patients with PMOS who had a first diagnosis of suicidal ideation within one year of being prescribed either metformin or semaglutide. Survival probabilities were estimated using the Kaplan-Meier method as implemented in the TriNetX Analytics platform. Hazard ratios were calculated using Cox proportional hazards regression. A total of 648 patients in Cohort 1 and 505 patients in Cohort 2 were excluded from the analysis because they had been diagnosed with suicidal ideation before their prescription. PMOS, polycystic metabolic ovarian syndrome

Cohort	Patients in cohort	Patients with outcome	Risk	Survival probability
Metformin	19,887	73	0.004	99.51%
Semaglutide	20,030	56	0.003	99.65%

The Kaplan-Meier survival curve analysis showed that patients in the metformin group had a 99.51% survival probability with respect to developing suicidal ideation, compared to 99.65% for the semaglutide group. Accordingly, the hazard ratio for suicidal ideation comparing metformin users to semaglutide users was 1.416 (95% CI: 1.000-2.006). The log-rank test was (χ² = 3.873, df = 1, *P* = 0.049) (Table 10).​​​​​

**Table 10:** Measures of comparison in suicidal ideation outcomes for patients with PMOS within one year of being prescribed either metformin or semaglutide. This table presents risk measures for suicidal ideation within one year, comparing patients with PMOS prescribed metformin versus semaglutide. Risk difference (RD) was calculated as the absolute difference in cumulative incidence between cohorts, risk ratio (RR) as the ratio of cumulative incidence, and odds ratio (OR) from the corresponding 2 × 2 contingency table. Hazard ratios (HRs) were estimated using a Cox proportional hazards regression model, while Kaplan-Meier survival curves were compared using the log-rank test. Corresponding *z*-statistics, chi-square statistics, *P*-values, and 95% confidence intervals (CIs) are reported for the appropriate analyses to indicate the statistical significance and precision of each estimate. Collectively, these measures quantify differences in the one-year risk of suicidal ideation between the metformin and semaglutide cohorts. PMOS, polycystic metabolic ovarian syndrome

Metric	Value	95% CI	z	*P*-value	Chi-squared	df
Risk difference	0.001	-0.000 to 0.002	1.540	0.124	-	-
Risk ratio	1.313	0.927-1.859	-	-	-	-
Odds ratio	1.314	0.927-1.862	-	-	-	-
Hazard ratio	1.416	1.000-2.006	-	0.247	1.343	1
Log-rank test	-	-	-	0.049	3.873	1

## Discussion

In this large real-world cohort of patients with PMOS, our findings suggest that semaglutide was not associated with a significantly greater risk of GAD or suicidal ideation compared with metformin; however, patients treated with semaglutide demonstrated a higher observed risk of MDD and insomnia compared with those treated with metformin. These findings support individualized treatment decisions while reinforcing the importance of ongoing mental health monitoring. Propensity score matching on age, race, ethnicity, and BMI allowed for improved comparability between treatment groups and reduced the likelihood of confounding. Additionally, use of the TriNetX platform enhanced the generalizability of our findings by incorporating data from a large, international network of healthcare institutions.

This study is unique in its focus on patients with PMOS, a population that is approximately 2.5 times more likely to develop depression compared with women without PMOS [1]. Semaglutide’s established efficacy as both a weight-management and glucose-regulating agent makes it a particularly well-suited therapeutic option for patients with PMOS. Clinical guidelines recommend weight loss as a first-line intervention for overweight and obese individuals with PMOS, as a 5%-10% reduction in body weight is associated with significant improvements in menstrual regularity and ovulatory function [4]. Consistent with these recommendations, a scoping review found that semaglutide use in patients with PMOS is associated with reductions in body weight, BMI, and waist circumference [11]. In light of the potential therapeutic benefits of semaglutide for patients with PMOS, studies examining its impact on mood disorders are essential to fully characterize its overall safety profile.

The dataset was propensity-matched on BMI, a novel methodological facet of this study. BMI is an independent risk factor for mood disorders [12], and GLP-1 receptor agonists are more frequently prescribed to individuals with higher BMI [13]. Matching on BMI was therefore critical to minimizing confounding related to both psychiatric vulnerability and prescribing patterns. This approach strengthens the interpretation of our findings by reducing the likelihood that observed differences in mood outcomes, including suicidal ideation, were driven by baseline weight-related risk rather than medication exposure.

Within this context, our findings can be situated within the broader and evolving literature examining the psychiatric safety of semaglutide, specifically in the PMOS patient population. Following the European Medicines Agency’s 2023 investigation into reports of suicidal ideation associated with GLP-1 receptor agonists, several studies have explored whether initiating semaglutide is associated with adverse mental health outcomes [14]. In fact, a separate TriNetX study found no association between semaglutide use and suicidal ideation when compared with other weight loss and antidiabetic medications [14]. Similarly, a meta-analysis evaluating the mental health effects of GLP-1 receptor agonists concluded that these medications do not increase the risk of suicidal ideation [15]. Our results extend these findings to a PMOS-specific population, reinforcing the absence of a detectable association between semaglutide use and development of GAD and suicidal ideation. However, given the evidence, there may be credence to changes in rates of MDD and insomnia at least in the first year of GLP-1 receptor agonist use.

Beyond suicidal ideation, GLP-1 receptor agonists have also been implicated in mood changes, anxiety, and insomnia in patient-reported data [9]. Arillotta et al.’s mixed-methods analysis of social media platforms identified several potential mechanisms underlying these observations. Weight loss itself may variably improve or worsen depressive symptoms, anxiety, and sleep quality. Additionally, GLP-1 receptor agonists influence inflammatory pathways, cortisol regulation, and thyroid hormone activity, which may contribute to improvements in depressive symptoms [9]. Obesity is a chronic inflammatory state that has been associated with increased mood symptoms [12]. Conversely, tightened glycemic control and fluctuations in blood glucose may negatively affect mood and increase vulnerability to depressive symptoms [9]. Reported increases in anxiety and insomnia may also be attributable to medication side effects such as nausea, fatigue, and gastrointestinal discomfort, with some users describing restlessness and difficulty sleeping while taking semaglutide [9]. Proposed neurobiological mechanisms, including modulation of GABAergic neurotransmission, may further contribute to anxiety and sleep disturbances [9]. These findings underscore the heterogeneity of patient experiences and highlight the role of individual susceptibility and treatment-related side effects. Importantly, our study suggests that psychiatric risk alone should not guide treatment decisions for women with PMOS.

This retrospective observational study provides comparative data on MDD, GAD, insomnia, and suicidal ideation among patients with PMOS treated with semaglutide or metformin. Given the substantial metabolic and reproductive benefits semaglutide may offer in this population, the absence of statistically significant adverse psychiatric outcomes in the GAD and suicidal ideation groups is clinically meaningful. Nevertheless, given the statistically significant, if minor, differences in the MDD and insomnia groups, treatment decisions should remain individualized, taking into account metabolic effects, fertility goals, side-effect profiles, patient preferences, cost, and access. Clinicians should continue to monitor mental health in all patients, particularly those with a history of psychiatric illness, regardless of the pharmacologic agent prescribed.

Strengths and limitations

This study utilized a large and diverse patient population drawn from the TriNetX electronic health record platform, which aggregates data from over 166 healthcare institutions worldwide. Use of standardized ICD-10 codes improved the consistency and objectivity of exclusion and inclusion criteria as well as the outcomes observed across institutions.

Several limitations should be acknowledged. As a retrospective analysis of medical data, causality cannot be established, and findings reflect associations rather than direct effects. Using an EHR system introduces the possibility of coding inaccuracies, misclassification of psychiatric diagnoses, and variability in documentation practices. The recent change from the term PCOS to PMOS in particular may lead to some coding variability that is not accounted for by the platform. The relatively short follow-up period may limit the detection of longer-term psychiatric outcomes. 

Medication adherence and correct usage of semaglutide and metformin could not be confirmed and may have influenced results. Although patients were required to have documented treatment for the full one-year study period, the database could not confirm whether patients took the medication as prescribed, including adherence to the prescribed dose or frequency. Additionally, medication discontinuation or changes in treatment may not have been captured if they were not documented within the database.

Additionally, despite propensity score matching, residual confounding from factors not included in the matching strategy could persist. Although patients with a pre-existing history of psychiatric illness were excluded, other factors such as diabetes and other metabolic comorbidities, concomitant medications, and healthcare utilization may influence treatment selection and mental health outcomes and were not included in the propensity-matching strategy. Other factors, such as social determinants of health, provider counseling, and medication cost, may also influence treatment selection and adherence but are not reliably captured in structured EHR data. The dynamic and multifactorial nature of mental health, weight change, and PMOS symptomatology may not be fully captured within structured EHR data.

## Conclusions

This study contributes to the growing body of evidence evaluating the psychiatric safety profile of GLP-1 receptor agonists by examining MDD, GAD, insomnia, and suicidal ideation in a population of women with PMOS. Among patients with PMOS treated with semaglutide compared with those treated with metformin, no statistically significant differences were observed in GAD or suicidal ideation. In contrast, statistically significant differences were observed for MDD and insomnia, with higher risk observed among patients taking semaglutide. These results suggest that psychiatric considerations alone should not preclude the use of semaglutide in PMOS management. Instead, shared decision-making should prioritize metabolic health, reproductive goals, side-effect profiles, and individual patient preferences. Ongoing mental health screening and follow-up remain essential for all patients with PMOS, regardless of treatment choice. Future research should focus on mechanistic studies exploring how weight loss, metabolic changes, and GLP-1 receptor activity may influence mood and sleep outcomes. Continued investigation will be critical to refining clinical guidelines and optimizing comprehensive care for individuals living with PMOS.
